# Elaborately Engineering a Self‐Indicating Dual‐Drug Nanoassembly for Site‐Specific Photothermal‐Potentiated Thrombus Penetration and Thrombolysis

**DOI:** 10.1002/advs.202104264

**Published:** 2021-11-21

**Authors:** Zhiqiang Zhao, Xuanbo Zhang, Hongyuan Zhang, Xinzhu Shan, Meiyu Bai, Zhe Wang, Fujun Yang, Haotian Zhang, Qiming Kan, Bingjun Sun, Jin Sun, Zhonggui He, Cong Luo

**Affiliations:** ^1^ Department of Pharmaceutics Wuya College of Innovation Shenyang Pharmaceutical University Shenyang Liaoning 110016 P. R. China; ^2^ School of Life Science and Biopharmaceutics Shenyang Pharmaceutical University Shenyang 110016 P. R. China

**Keywords:** antiplatelet, dual‐drug nanoassembly, photothermal thrombolysis, site‐specific synergistic thrombolysis, thrombus penetration

## Abstract

Thrombotic cardio‐cerebrovascular diseases seriously threaten human health. Currently, conventional thrombolytic treatments are challenged by the low utilization, inferior thrombus penetration, and high off‐target bleeding risks of most thrombolytic drugs, resulting in unsatisfactory treatment outcomes. Herein, it is proposed that these challenges can be overcome by precisely integrating the conventional thrombolytic strategy with photothermal therapy. After co‐assembly engineering optimization, a fibrin‐targeting peptide‐decorated nanoassembly of DiR (a photothermal probe) and ticagrelor (TGL, an antiplatelet drug) is prepared for thrombus‐homing delivery, abbreviated as FT‐DT NPs. The elaborately engineered nanoassembly shows multiple advantages, including simple preparation with high drug co‐loading capacity, synchronous delivery of two drugs with long systemic circulation, thrombus‐targeted accumulation with self‐indicating function, as well as photothermal‐potentiated thrombus penetration and thrombolysis with high therapeutic efficacy. As expected, FT‐DT NPs not only show bright fluorescence signals in the embolized vessels, but also perform photothermal/antiplatelet synergistic thrombolysis in vivo. This study offers a simple and versatile co‐delivery nanoplatform for imaging‐guided photothermal/antiplatelet dual‐modality thrombolysis.

## Graphical abstract

To achieve site‐specific photothermal‐potentiated thrombus penetration and thrombolysis, a carrier‐free nanoassembly is self‐aggregated by DiR and TGL. The dual‐drug nanoassembly demonstrates multiple advantages, including simple preparation with high drug co‐loading capacity, synchronous co‐delivery with long systemic circulation, thrombus‐targeted accumulation with self‐indicating function, as well as photothermal‐potentiated thrombus penetration and thrombolysis with high security.

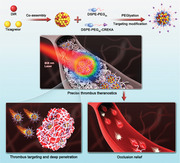

## 1 Introduction

Thrombotic cardio‐cerebrovascular disorders represent one of the most serious diseases with high morbidity and mortality worldwide, such as stroke, myocardial infarction, and venous thromboembolism.^[^ ^1^ ^]^ Thrombus refers to small blood clots consisted of water‐insoluble fibrins, activated platelets, white blood cells, or erythrocytes.^[^ ^2^ ^]^ Generally, thrombosis could be divided into two types, namely arterial thrombosis (AT) and venous thrombosis (VT). The AT involves multiple stages, including: i) rupture of an atherosclerotic plaque; ii) recruitment, aggregation, and activation of platelets; iii) activation of the coagulation cascade; and iv) generation and aggregation of fibrin.^[^ ^3^ ^]^ In the other case, hypercoagulable state, vascular wall injury and circulatory stasis have been found to be able to trigger the VT.^[^ ^4^ ^]^ Abnormal thrombosis usually leads to vessel occlusion, blood flow blockage and ischemia damages to organs (e.g., heart, brain, and lung), resulting in life‐threatening diseases or permanent disability.^[^ ^5^ ^]^

Given the high morbidity and mortality of thrombotic diseases, rational design of novel theranostic strategies with high efficiency and security has long been a high priority for clinical antithrombotic treatment. Fibrinolytics, anticoagulants, and antiplatelet agents represent the commonly used antithrombotic drugs in clinics.^[^ ^3^ ^]^ Among them, fibrinolytic drugs are usually used for the treatment of acute thrombotic diseases, such as tissue plasminogen activator (tPA).^[^ ^6^ ^]^ Moreover, a variety of anticoagulants and antiplatelet agents have been developed for the prevention and treatment of thromboembolic disorders.^[^ ^7^ ^]^ Among them, ticagrelor (TGL) shows potent antiplatelet activity by reversibly antagonizing the P2Y_12_ receptor and inhibiting the adenosine diphosphate (ADP)‐induced platelet aggregation.^[^ ^8^ ^]^ Disappointingly, conventional antithrombotic agents have long been criticized for unsatisfactory therapeutic effects with high bleeding risks, due to off‐target biodistribution, inferior thrombus penetration and narrow therapeutic windows.^[^ ^9^ ^]^ Particularly, the insufficient thrombus penetration of antithrombotic drugs has seriously impeded the clinical therapeutic effect.^[^ ^10^ ^]^ Recently, several clot‐penetrating drug delivery strategies have been developed to address this challenge, such as ultrasound‐ and gas‐facilitated thrombus penetration.^[^ ^10^ ^]^ Nevertheless, these invasive and mechanical penetration strategies have major drawbacks, which greatly limited their clinical applications, such as complex manipulation, inconvenient real‐time monitoring, potential damage on the surrounding vascular tissues, and poor patient compliance.^[^ ^10^ ^]^

In addition to drug therapy, photothermal photosensitizers (PSs) have also been extensively investigated for near infrared (NIR) thrombus imaging and hyperthermal thrombolysis.^[^ ^11^ ^]^ There is growing evidence showing that a sharp temperature rise in thrombus tissues effectively loosens the clots via destroying the non‐covalent interactions of fibrins, which not only exerts hyperthermal thrombolysis but also facilitates the deep penetration of antithrombotic drugs in thrombi.^[^ ^12^ ^]^ Notably, deep‐thrombus photothermal penetration represents a safe and non‐invasive clot‐penetrating approach. Nevertheless, despite the distinct advantages of convenient imaging, deep‐thrombus penetration and precise localizing treatment, photothermal intervention alone is usually not potent enough for thrombus eradication. Moreover, photothermal thrombolysis has been found to tend to recur post‐therapy, significantly restricting its clinical applications.^[^ ^12^ ^]^

To deal with the unsatisfactory therapeutic outcomes of either conventional antithrombotic drugs or photothermal thrombolysis, combination therapy has shown remarkable advantages in thrombus treatment.^[^ ^13^ ^]^ However, it remains challenging to precisely co‐deliver multiple therapeutic drugs into thrombi.^[^ ^14^ ^]^ With the burgeoning of biomedical nanotechnology, nanoparticulate drug delivery system (nano‐DDS) has shown noteworthy advantages in antithrombotic drug delivery, mainly including:^[^ ^15^ ^]^ i) improving the physicochemical properties of antithrombotic drugs; ii) prolonging the systemic circulation time; iii) realizing the site‐specific drug delivery; and iv) reducing the off‐target bleeding risks. More importantly, multiple theranostic agents could be co‐encapsulated into one nanosystem for multimodal theranostics.^[^ ^16^ ^]^ Despite the many advantages of co‐delivery nano‐DDS, co‐encapsulation of two or more drugs in conventional nanocarriers has long been criticized for low drug co‐loading efficiency, inconvenient dose ratio adjustment, poor colloidal stability, and potential drug leakage.^[^ ^7^ ^]^ Additionally, the potential toxicity of carrier materials has been widely regarded as a leading obstacle hindering the clinical translation of nanomedicines.^[^ ^18^ ^]^ Therefore, there is an urgent need to develop novel co‐delivery nanosystems with the characteristics of facile fabrication, high efficiency, and low toxicity.

**Figure 1:**
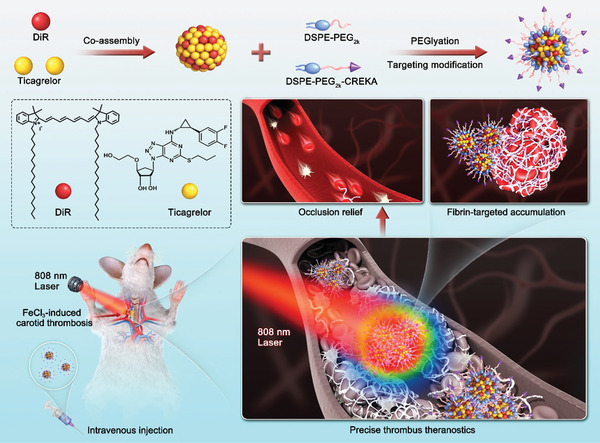
Schematic representation of DiR/TGL co‐assembly and precisely imaging‐guided thrombolysis. The carrier‐free nanoassembly was self‐aggregated by DiR and TGL without the assistance of any polymer. The dual‐drug co‐assembly was modified with DSPE‐PEG_2K_ and DSPE‐PEG_2K_‐CREKA for long circulation in blood and fibrin‐targeting accumulation in thrombus. Self‐indicating precise delivery and potent anti‐thrombus activity were observed in an FeCl_3_‐induced carotid thrombosis rat model.

In the present study, we hypothesized that a precise combination of photothermal probes and traditional antiplatelet drugs would be a promising antithrombotic strategy. Particularly, thrombus‐localizing temperature rise not only exerts hyperthermal thrombolysis effect, but also facilitates the deep thrombus penetration of antithrombotic drugs, hopefully realizing photothermal‐amplified antithrombotic effect. Furthermore, antiplatelet drugs are able to suppress thrombosis by inhibiting platelet activation and aggregation, which helps prevent secondary embolism caused by the fragments of photothermal thrombolysis. To test our hypothesis, a carrier‐free nanosystem self‐assembled by TGL and 1,1′‐dioctadecyl‐3,3,3′,3′‐tetramethylindotricarbocyanine iodide (DiR) was elaborately fabricated based on an interesting discovery of dual‐drug co‐assembly (**Figure** **1**). Multiple intermolecular interactions and forces were found to drive the co‐assembly process of DiR and TGL. In order to improve the pharmacokinetic behaviors and to endow the nanoassembly with thrombus‐targeting capacity, 1,2‐distearoyl‐sn‐glycero‐3‐phosphoethanolamine‐*N*‐[methoxy(polyethyleneglycol)‐2000] (DSPE‐PEG_2K_) and Cys‐Arg‐Glu‐Lys‐Ala (CREKA, a fibrin‐targeted pentapeptide)‐conjugated DSPE‐PEG_2K_ (DSPE‐PEG_2K_‐CREKA) were utilized to decorate on the surface of NPs. The fibrin‐targeting DiR/TGL NPs (FT‐DT NPs) showed high site‐specific accumulation, deep thrombus penetration, efficient photothermal conversion, as well as potent photothermal thrombolysis effect. As expected, FT‐DT NPs not only revealed bright fluorescence signals in the embolized vessels, but also exerted photothermal/antiplatelet synergistic antithrombotic activity in a FeCl_3_‐induced carotid thrombosis rat model. This is the first time that antithrombotic drugs and photothermal probes are ingeniously integrated into a facile carrier‐free nanosystem for self‐indicating multimodal thrombus therapy.

## 2 Results and Discussion

### 2.1 Precise Fabrication and Characterization of a Dual‐Drug Nanoassembly

**Figure 2:**
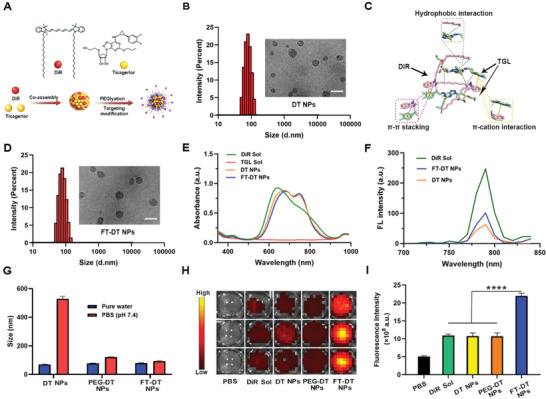
Fabrication and characterization of DT NPs and FT‐DT NPs. A) Schematic representation of the co‐assembly process. B) Particle size and TEM images of DT NPs (scale bar represents 100 nm). C) Computational docking simulation results of DiR and TGL in the aqueous medium. D) Particle size and TEM images of FT‐DT NPs (scale bar represents 100 nm). E) US spectra of DiR Sol, TGL Sol, DT NPs, and FT‐DT NPs. F) Fluorescence spectra of DiR Sol, DT NPs, and FT‐DT NPs. G) Colloidal stability of DT NPs, PEG‐DT NPs, and FT‐DT NPs incubated in pure water or PBS (pH 7.4) for 30 min. H) In vitro fluorescence signals in artificial blood clots. I) Quantitative analysis of fluorescence intensity in artificial blood clots (*n* = 3), ****p < 0.0001.

Interestingly, DiR and TGL molecules were found to readily co‐assemble into NPs through a simple one‐step nano‐precipitation, without the assistance of any carrier material (**Figure** **2**A; Figure S1, Supporting Information). The co‐assembly formulation was optimized by evaluating the mean diameters and polydispersity index (PDI) of the non‐PEGylated nanoassemblies (DT NPs) with different mass ratios (3:1, 2:1, 1:1, 1:2, and 1:3) of DiR and TGL. As shown in Table S1, Supporting Information, DT NPs with a mass ratio of 1:1 stood out as the optimal nanoassembly with favorable particle size (74.96 ± 0.42 nm) and PDI (0.14 ± 0.05). Moreover, DT‐NPs showed uniform spherical nanostructures (Figure 2B) with a slight positive Zeta potential (17.50 ± 0.09 mV, Table S2, Supporting Information), which should be ascribed to the quaternary amine group of DiR (Figure 2A). Thus, the nanoassembly with an optimal mass ratio of DiR and TGL (1:1) was selected to carry out the subsequent experiments.

We then explored the co‐assembly mechanisms of DiR and TGL. As shown in Figure 2A, the aromatic groups in both DiR and TGL molecules might play essential roles in the co‐assembly process by forming intermolecular *π*–*π* stacking and *π*–cation interactions. Moreover, hydrophobic forces have also been commonly recognized as a vital force driving the assembly of water‐insoluble compounds.^[^ ^19^ ^]^ To figure out the existing intermolecular interactions between DiR and TGL, computational docking simulation was utilized to illustrate the co‐assembly mechanisms in DT‐NPs. As shown in Figure 2C, a trimer cluster was found to be formed by one DiR molecule and two TGL molecules. Notably, the mass ratio of DiR (mw = 1013.4) and TGL (mw = 522.6) in the trimer cluster was very close to 1:1, which was in well consistence with the results of nanoassembly optimization (Table S1, Supporting Information). Notably, multiple interactions and forces were found in the trimer cluster, including hydrophobic force, *π*–*π* stacking, as well as *π*–cation interaction (Figure 2C).

To endow DT‐NPs with long systemic circulation and thrombus‐homing ability, a fibrin‐specific peptide was conjugated with DEPE‐PEG_2K_ to obtain DEPE‐PEG_2K_‐CREKA (Figure S2, Supporting Information). The successful synthesis of DEPE‐PEG_2K_‐CREKA was confirmed by mass spectrum (MS, Figure S3, Supporting Information). Then, DEPE‐PEG_2K_‐CREKA and DSPE‐PEG_2K_ were decorated onto the surface of DT NPs to obtain a PEGylated fibrin‐homing nanoassembly (FT‐DT NPs). As shown in Figure 2D and Table S2, Supporting Information, the mean diameter of DT‐NPs slightly increased after modification. Notably, FT‐DT NPs showed a positive‐negative change in zeta potential from 17.50 ± 0.09 mV to −16.10 ± 0.35 mV, probably due to the phosphate groups in DSPE‐PEG_2K_ and DEPE‐PEG_2K_‐CREKA. Significantly, the drug loading rates of DiR and TGL in FT‐DT NPs were up to 37.5 wt%, respectively (Table S2, Supporting Information). The high co‐loading capacity of dual‐drug nanoassemblies certainly benefits synergistic antithrombotic therapy.

Moreover, the UV absorbance spectra of DT NPs and FT‐DT NPs appeared a slight red‐shift when compared to that of DiR Sol (Figure 2E), suggesting the existence of intermolecular *π*–*π* stacking interaction between DiR and TGL.^[^ ^18^ ^]^ TGL showed no obvious UV absorption in the wavelength from 500 to 900 nm (Figure 2E). Notably, the peak position of DT‐NPs and FT‐DT NPs was very similar to DiR Sol in the fluorescence spectra, while the fluorescence intensity of nanoassembly was weaker than that of DiR Sol (Figure 2F). The attenuated fluorescence signals should be attributed to the aggregation caused quenching (ACQ) effect of most fluorescent agents in the state of aggregation.^[^ ^20^ ^]^ Notably, the fluorescence intensity of FT‐DT NPs was stronger than that of the naked DT NPs, probably due to the larger molecule distance between DiR molecules in FT‐DT NPs after insertion of the hydrophobic DSPE section in the nanoassembly.

### 2.2 Colloidal Stability and In Vitro Thrombus‐Targeting Capacity

As previously mentioned, a fibrin‐targeting peptide (CREKA) was utilized to endow FT‐DT NPs with thrombus‐targeting ability. In order to compare the contribution of PEGylation and CREKA modification to the colloidal stability and thrombus‐targeting capacity of nanoassemblies, the PEGylated non‐targeting NPs (PEG‐DT NPs) were prepared (Figure S4 and Table S2, Supporting Information). As shown in Figure 2G, both PEG‐DT NPs and FT‐DT NPs exhibited good colloidal stability after incubation with PBS (pH 7.4) for 30 min when compared with the non‐PEGylated DT NPs, suggesting the important role of PEGylation modification in resisting the salting‐out effect. To further explore the colloidal and co‐loading stability of PEGylated nanoassemblies in a simulated physiological environment, PEG‐DT NPs and FT‐DT NPs were incubated in PBS (pH 7.4) containing 10% FBS for 12 h at 37 °C. As shown in Figures S5 and S6, Supporting Information, both PEG‐DT NPs and FT‐DT NPs exhibited good colloidal stability in a simulated physiological environment, with no significant change in particle size. More importantly, more than 95% of the drugs (DiR and TGL) could be stably maintained in the nanoassemblies incubated in PBS (pH 7.4) containing 10% FBS for 12 h at 37 °C, probably due to the multiple intermolecular forces existed in the nanoassemblies between DiR and TGL (Figure 2C). These results suggested that the dual‐drug nanoassemblies had good colloidal and co‐loading stability in the present of salts and proteins. Moreover, as shown in Figure S7, Supporting Information, DT NPs, PEG‐DT NPs and FT‐DT NPs showed good long‐term stability at 4 °C of under dark conditions for one week, with no significant changes in particle sizes and surface charge.

Furthermore, an artificial blood colt was prepared to explore the thrombus‐targeting capacity of the PEGylated fibrin‐targeting FT‐DT NPs.^[^ ^21^ ^]^ The fluorescence signals in artificial blood colts were detected after incubation with PBS (control), DiR Sol, DT NPs, PEG‐DT NPs, and FT‐DT NPs, respectively. As shown in Figure 2H and 2I, FT‐DT NPs exhibited much stronger fluorescence signals in clots than that of DiR Sol, DT NPs and PEG‐DT NPs, indicating the important role of CREKA peptide in thrombus‐targeting drug accumulation. Thus, FT‐DT NPs showed distinct superiority over DT NPs and PEG‐DT NPs in terms of colloidal stability and thrombus‐targeting capacity.

### 2.3 In Vitro Photothermal Conversion Efficiency

**Figure 3:**
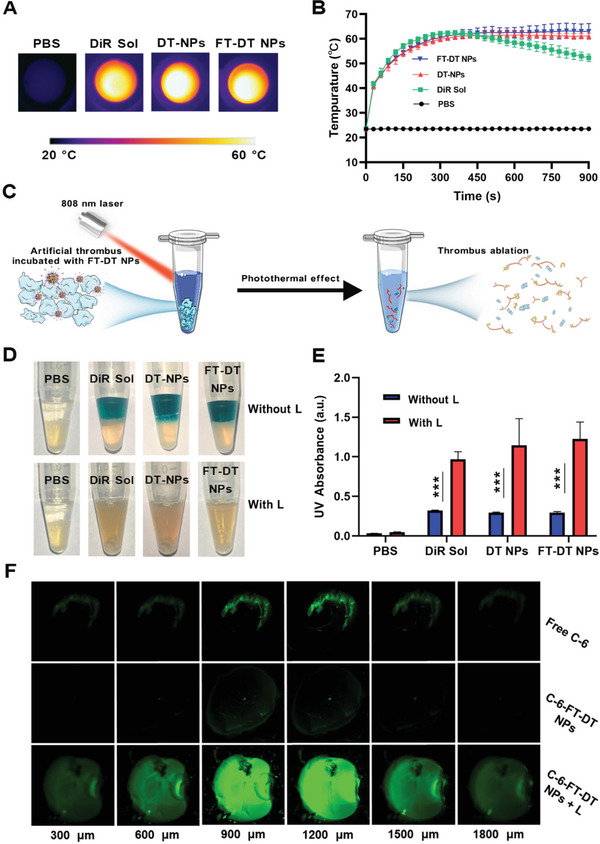
In vitro photothermal conversion and photothermal thrombolysis efficiency. A,B) In vitro photothermal conversion efficiency (*n* = 3). C) Scheme of photothermal ablation of artificial clots. D) Appearance photos of in vitro photothermal thrombolysis effects. E) The UV absorption (450 nm) of fibrins dissolved in the supernatants after treatments (*n* = 3). F) Confocal imaging of small red thrombus treated with free C‐6, C‐6‐FT‐DT NPs, or C‐6‐FT‐DT NPs + L for 30 min. Laser irradiation condition (808 nm, 2.0 W cm^−2^, 10 min), ***p<0.001.

DiR has been widely used as a photothermal agent for photothermal therapy (PTT). As previously discussed, the fluorescence intensity of both DT NPs and FT‐DT NPs was weaker than that of DiR Sol due to the ACQ effect (Figure 2F). So, we wondered whether the co‐assembly process also exerted significant influence on the photothermal conversion efficiency of DiR. As shown in **Figure** **3**A and 3B, DiR Sol, DT NPs and FT‐DT NPs showed rapid rise in temperature up to ≈60 °C within 200 s under laser irradiation (808 nm), while the temperature of PBS barely changed under the same conditions. Notably, the temperature of DiR Sol showed a downward trend (from 60 to 50 °C) starting at 500 s, while DT NPs and FT‐DT NPs showed a persistent hyperpyrexia feature under the same conditions. The comparable photothermal conversion efficiency and persistent hyperpyrexia characteristics of nanoassembly might be attributed to the “supramolecular photothermal effects,” namely photosensitizers usually have higher photothermal conversion ability with photoluminescence quenching in the state of aggregation than that in the individual molecular states.^[^ ^22^ ^]^ These results suggested that nanoassembly performed efficient photothermal conversion, which could be beneficial to the photothermal thrombolysis.

### 2.4 In Vitro Light‐Triggered and Shear‐Responsive Drug Release

The antithrombotic efficiency of FT‐DT NPs heavily depends on the release profile of TGL from the dual‐drug nanoassemblies. We supposed that laser irradiation could promote TGL release from FT‐DT NPs. The release behaviors of TGL from FT‐DT NPs with/without laser irradiation were explored. As shown in Figure S8, Supporting Information, around 50% of TGL could be continually released from FT‐DT NPs in 12 h. Interestingly, laser irradiation could significantly facilitate the release of TGL from FT‐DT NPs, with about 80% of TGL release under the same conditions. Laser‐facilitated TGL release from the FT‐DT NPs should be attributed to the disintegration of the nanoassemblies following the photobleaching of DiR, since the intermolecular interactions between DiR and TGL would be weakened or even disappeared after the photobleaching destroy of DiR molecules. As shown in Figure S9, Supporting Information, there was a distinct change in the morphology of FT‐DT NPs after laser irradiation, suggesting the laser‐triggered disassembly of FT‐DT NPs.

In addition, it has been found that the reduction in the lumen of the vessel caused by large thrombus formation usually increases the local shear stress by 1–2 orders of magnitude.^[^ ^15^ ^]^ We then investigated the influence of agitation shear force on the release of TGL from FT‐DT NPs. Slight agitation shear force was utilized to simulate the high hemodynamic shear stress at the thrombus site. As shown in Figure S10, Supporting Information, it turned out that agitation shear force significantly facilitated the release of TGL from FT‐DT NPs, which might be attributed to the influence of agitation shear force on the intermolecular interactions in the nanoassemblies. These results suggested that both the photobleaching effect and mechanical stress exerted significant impacts on the nanostructure of FT‐DT NPs, due to the destructive effects of laser and shear force on the intermolecular forces between DiR and TGL. As shown in Figures S8 and S10, Supporting Information, both laser irradiation and agitation shear force distinctly promoted TGL release from the nanoassemblies. Such a laser/shear dual‐sensitive drug release characteristic within thrombus site could certainly enhance thrombus therapy effect with low systemic toxicity.

### 2.5 In Vitro Photothermal Thrombolysis and Deep Thrombus Penetration

Encouraged by the excellent photothermal conversion efficiency of FT‐DT NPs, we further explored the in vitro thrombolysis activity. Without treatment, the artificial blood clots at the bottom of the tubes remain intact even after turning the tube over several times. By contrast, the blood clots could be destroyed after thrombolytic treatment, and the components in the blood clot would be dispersed in the medium under the same conditions (Figure 3C). As expected, DiR Sol, DT‐NPs, and FT‐DT NPs exhibited photothermal thrombolysis effects under laser irradiation (808 nm, 2.0 W cm^−2^, 15 min). As shown in Figure 3D, the dark‐colored supernatants came from the color of DiR without laser treatment, and the lower half of the tubes were the blood clots. Under laser irradiation, the disappearance of the dark‐colored supernatants was observed, due to the photobleaching of DiR (Figure 3D). Moreover, it can be clearly seen that the blood clots were destroyed by DiR Sol, DT‐NPs, and FT‐DT NPs under laser irradiation, while laser irradiation showed almost no influence on the blood clots in the PBS group (Figure 3D). The absorption of fibrins dissolved in the supernatants was further determined by UV spectrophotometer. As shown in Figure 3E, DiR Sol, DT‐NPs, and FT‐DT NPs showed excellent ability to lyse the fibrins in blood clots under laser irradiation, indicating the exact effect of photothermal thrombolysis.

**Figure 4:**
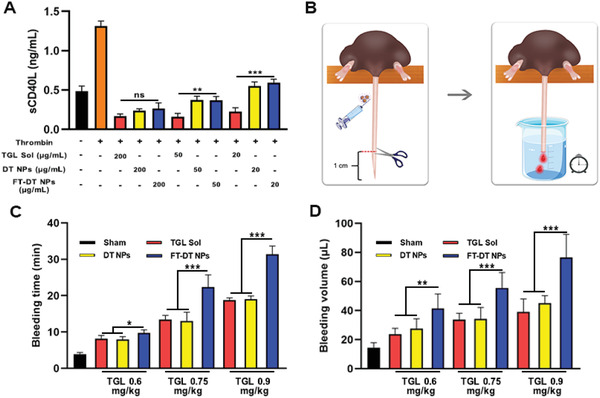
In vitro and in vivo antiplatelet activities. A) The expression levels of sCD40L in the activated platelets after treatments, in which thrombin was utilized to activate the platelets (*n* = 3). B) A schematic diagram of tail bleeding assay. C,D) The bleeding time and volume in the tail bleeding assay (*n* = 5), *p < 0.05, **p < 0.01, ***p < 0.001, ns: no significance.

**Figure 5:**
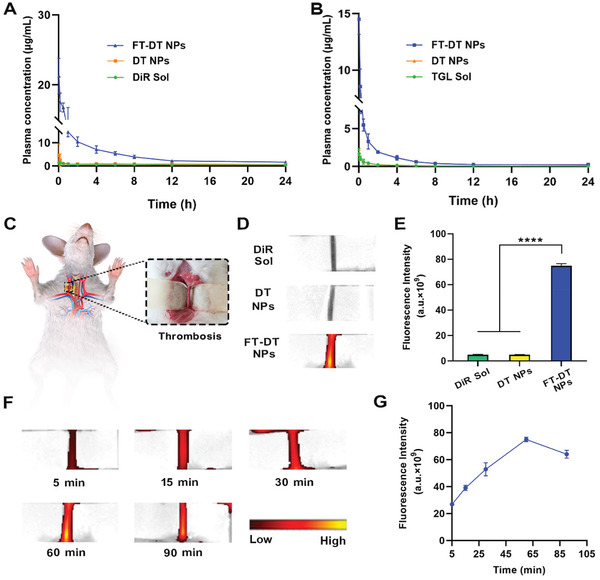
Self‐indicating thrombus‐specific fluorescence imaging. A) Concentration‐time curves of DiR Sol, DT NPs, and FT‐DT NPs following intravenous injection with an equivalent dose DiR of 5 mg kg^−1^ (*n* = 5). B) Concentration‐time curves of TGL Sol, DT NPs, and FT‐DT NPs following intravenous injection with an equivalent dose TGL of 5 mg kg^−1^ (*n* = 5). C) Scheme of establishing an FeCl_3_‐induced rat carotid arterial thrombosis model. D) In vivo fluorescence images of the carotid arterial thrombotic vessels at 1 h post‐injection of DiR Sol, DT NPs, or FT‐DT NPs. E) Quantitatively analyzing the fluorescence intensity of the carotid arterial thrombotic vessels at 1 h post‐injection of DiR Sol, DT NPs, or FT‐DT NPs (*n* = 5). F) Fluorescence images of the carotid arteries thrombotic vessels at different time points post‐injection of FT‐DT NPs. G) Quantitatively analyzing the fluorescence intensity of the carotid arterial thrombotic vessels at different time points post‐injection of FT‐DT NPs (*n* = 5), ****p < 0.0001.

**Figure 6:**
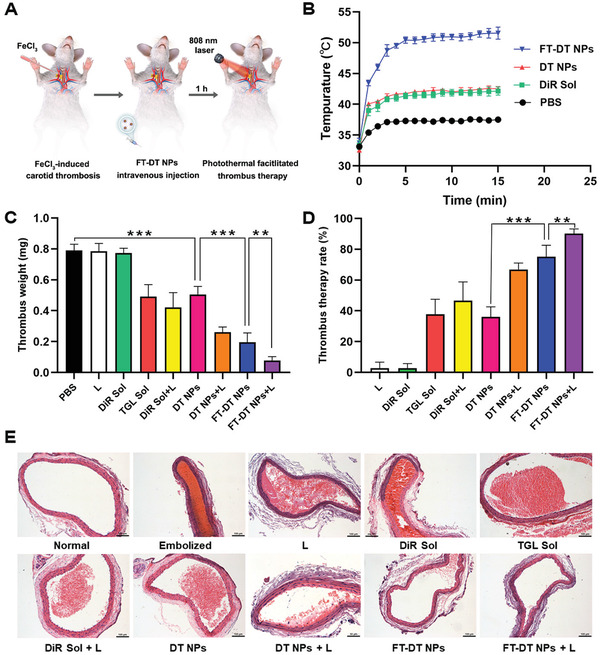
In vivo photothermal conversion efficiency and site‐specific photothermal‐amplified thrombolysis effect in a FeCl_3_‐induced rat carotid arterial thrombosis model. A) Treatment scheme. B) Temperature changing curves of thrombus‐specific location irradiated by 808 nm laser (2.0 W cm^−2^, 0–15 min) (*n* = 5). C) Thrombus dry weight in the embolized vessel treated with different formulations with/without laser. D) Thrombus therapy rate after treatments with different formulations with/without laser treatment (*n* = 6). E) Sections of carotid artery vessels stained with H&E. Scale bar represents 100 µm, **p < 0.01 and ***p < 0.001.

It has been found that the local thermal effect of PTT significantly enhances the tissue penetration ability of nanomedicines in the blood clots.^[^ ^12^ ^]^ Based on this rationale, the thermal effect generated by DiR under irradiation would also facilitate the deep penetration and retention of FT‐DT NPs into the thrombi. To evaluate the penetration and retention capacity of FT‐DT NPs, a small red thrombus model was established, and coumarin‐6 (C‐6) was used to label FT‐DT NPs (C‐6‐FT‐DT NPs). As illustrated in Figure 3F, C‐6‐FT‐DT NPs without irradiation showed poor thrombus penetration ability, even more inefficient than that of free C‐6. By contrast, C‐6‐FT‐DT NPs with laser irradiation showed extremely strong fluorescent signals in the interior regions of the thrombus (Figure 3F), suggesting the photothermal‐facilitated deep thrombus penetration ability of FT‐DT NPs.

### 2.6 In Vitro and In Vivo Antiplatelet Activities

Given the potent photothermal thrombolysis activity of FT‐DT NPs under laser irradiation (Figure 3D,E), it is necessary to further evaluate the antiplatelet activity. It has been widely recognized that the overexpressed sCD40L on the activated platelets plays an important role in thrombosis and inflammatory through interacting with the CD40 protein on endothelial cells or immune cells.^[^ ^23^ ^]^ Thus, the expression of sCD40L in the activated platelets is usually utilized as an indicator for the evaluation of antiplatelet activity. As shown in **Figure** **4**A, the sCD40L level was significantly elevated after the activation of platelets by thrombin, with a nearly threefold increasement of sCD40L when compared with normal platelets (saline group). TGL Sol significantly downregulated the sCD40L levels (Figure 4A), suggesting the potent antiplatelet activity of TGL. Notably, DT NPs and FT‐DT NPs demonstrated comparable antiplatelet activities with TGL Sol at a high TGL concentration of 200 µg mL^−1^, due to the saturation binding of P2Y_12_ receptors on the platelet membrane with TGL released from the nanoassemblies. Notably, the antiplatelet effects of DT NPs and FT‐DT NPs dramatically decreased along with the decrease of incubation concentrations from 200 to 20 µg mL^−1^, while the antiplatelet activity of TGL Sol showed no apparent variation under the same conditions (Figure 4A). Even so, both DT NPs and FT‐DT NPs could effectively inhibit thrombin‐induced platelet activation at a low incubation concentration of TGL (20 µg mL^−1^), with sCD40L level down to near normal level. These results indicated that formulating TGL into a dual‐drug nanoassembly still exerted good antiplatelet effect.

We then further evaluated the in vivo antiplatelet activity of FT‐DT NPs through a classical mouse tail bleeding assay (Figure 4B).^[^ ^24^ ^]^ Briefly, the mouse tails were amputated to initiate bleeding at 2 h post intravenous administration of TGL Sol, DT NPs, or FT‐DT NPs. The bleeding time and volume were recorded as the evaluating indicators of antiplatelet ability. As shown in Figure 4C, rapid thrombosis and hemostasis occurred in the sham group within 5 min due to the inherent hemostatic function of the mouse. By contrast, TGL Sol and DT NPs significantly prolonged the bleeding time and increased the bleeding volume when compared with the sham group, owing to the potent antiplatelet activity of TGL. Notably, FT‐DT NPs exhibited distinct superiority over TGL Sol and DT‐NPs in terms of anticoagulation (Figure 4C), suggesting the excellent in vivo antiplatelet activity of the PEGylated fibrin‐homing nanoassembly. Similar trends was found in the bleeding volume (Figure 4D), and the increasement in bleeding time and bleeding volume presented a dose‐dependent manner. We supposed that the excellent in vivo antiplatelet efficacy of FT‐DT NPs should be primarily ascribed to its thrombus‐targeting ability. To verify this, the thrombus‐targeting capacity of FT‐DT NPs was investigated by detecting the fluorescence signals in the amputated tail after soaking with DT‐NPs and FT‐DT NPs for 3 min. As shown in Figure S11, Supporting Information, the FT‐DT NPs‐soaked mouse tails showed much stronger fluorescence signal than that of the naked DT‐NPs‐treated tails. These results illustrated that FT‐DT NPs with excellent thrombus‐targeting ability revealed potent antiplatelet activities in vitro and in vivo.

### 2.7 In Vivo Pharmacokinetics and Self‐Indicating Thrombus‐Specific Drug Delivery

The systemic circulation time of therapeutic agents exerts significant impacts on the treatment outcomes of diseases, especially for the lesions located in blood vessels. Particularly, we expected that PEGylation and CREKA peptide modifications on DT NPs would not only prolong the circulation time in the blood, but also facilitate the thrombus‐specific accumulation of NPs. In this section, Sprague‐Dawley rats were utilized to explore the pharmacokinetics of DiR Sol, TGL Sol, DT NPs, and FT‐DT NPs. The plasma concentrations of DiR were determined by fluorescence analysis, and the plasma concentrations of TGL were measured by UPLC‐MS‐MS. As shown in **Figure** **5**A,B and Tables S3,S4, Supporting Information, DiR Sol and TGL Sol were quickly cleared from the blood after intravenous injection. Similarly, DT NPs with poor stability also revealed poor pharmacokinetic behavior. In addition to poor colloidal stability (Figure 2G), the positively charged DT NPs (Table S2, Supporting Information) would interact with the negatively charged proteins and other biomacromolecules in the blood, which could not be conducive to the favorable systemic circulation after intravenous injection. As expected, FT‐DT NPs significantly extended the circulation time of both DiR and TGL in the blood (Figure 5A,B), which should be attributed to good colloidal stability and the stealth effect of PEGylation decoration. These results suggested that the co‐assembly of DiR and TGL distinctly changed their pharmacokinetic behaviors, and PEGylation modification on the nanoassemblies contributed to extending the circulation time of both DiR and TGL in the blood. More importantly, the plasma concentration‐time curves of DiR and TGL in FT‐DT NPs showed a very similar variation trend (Figure 5A,B), which should be ascribed to the excellent stability of the PEGylated dual‐drug nanoassemblies.

We then studied the self‐indicating thrombus‐targeting efficiency of FT‐DT NPs in an FeCl_3_‐induced arterial thrombosis rat model (Figure 5C). As previously mentioned, a fibrin‐targeting peptide (CREKA) was utilized to endow FT‐DT NPs with thrombus‐targeting ability. As shown in Figure 5D,E, the FT‐DT NPs group showed strong fluorescence signals in the obstructed vessels at 1 h post intravenous injection. By contrast, only very weak fluorescence signals were observed in both DiR Sol and DT‐NPs groups. These results suggested the excellent thrombus‐specific delivery feature of FT‐DT NPs, which should be attributed to the prolonged circulation time in the blood and the decoration of CREKA peptide on NPs. Furthermore, we examined the fluorescence signal changes in the blood clots treated with FT‐DT NPs within 90 min to figure out the laser irradiation time in the in vivo thrombolytic therapy schedule. As shown in Figure 5F,G, the fluorescence signals in the clots climbed up and then declined, and the strongest fluorescence signal was found at 1 h post‐injection of FT‐DT NPs.

### 2.8 Site‐Specific Photothermal‐Amplified Thrombolysis

The favorable colloidal stability, photothermal conversion efficiency, antiplatelet activity, pharmacokinetic behavior, as well as the thrombus‐targeting ability of FT‐DT NPs make it a promising nanomedicine for clinical thrombus therapy. The in vivo photothermal‐amplified thrombolysis activity of FT‐DT NPs was evaluated in an FeCl_3_‐induced rat carotid artery thrombosis model. As shown in **Figure** **6**A, PBS, DiR Sol, TGL Sol, DT NPs, and FT‐DT NPs were intravenously administrated to the rats for one injection at an equivalent dose of 5 mg kg^−1^ (DiR) and/or 5 mg kg^−1^ (TGL). The laser‐treated groups (DiR Sol, DT NPs, and FT‐DT NPs) received thrombus‐localized irradiation (808 nm, 2.0 W cm^−2^) for 15 min at 1 h post‐administration.

First, we evaluated the in vivo photothermal efficiency of FT‐DT NPs in an FeCl_3_‐induced rat carotid arterial thrombosis model under laser irradiation (808 nm). As shown in Figure 6B and Figure S12, Supporting Information, FT‐DT NPs, with remarkable temperature elevation at thrombus site (over 50 °C), demonstrated much higher in vivo photothermal conversion efficiency than that of DiR Sol (around 43 °C) and DT NPs (around 43 °C). Although FT‐DT NPs showed comparable in vitro photothermal conversion efficiency and photothermal thrombolysis capacity with DiR Sol and DT‐NPs (Figure 3), the favorable pharmacokinetic behavior and in vivo thrombus‐targeting ability of FT‐DT NPs (Figure 5A,D) contributed to excellent in vivo photothermal efficiency.

Then, the site‐specific photothermal‐amplified thrombolysis efficacy of FT‐DT NPs was further investigated. As shown in Figure 6C–E, laser irradiation only and DiR Sol without laser showed almost no therapeutic effect. Moreover, TGL Sol and DiR Sol with laser only demonstrated moderate thrombolysis effect, due to the rapid clearance and off‐target distribution of free drugs in the body. Owing to the inferior pharmacokinetic property and thrombus‐targeting capacity, DT NPs without laser irradiation showed comparable thrombolysis effect with TGL Sol. Notably, laser irradiation enhanced the antithrombotic effect of DT NPs to a certain extent, suggesting the synergy effect of TGL and DiR. As expected, FT‐DT NPs, even without laser treatment, revealed a potent thrombolysis effect with a therapeutic rate over 75% (Figure 6D), which should be attributed to the significant improvement of thrombus‐specific accumulation of TGL. More importantly, the antithrombotic efficacy of FT‐DT NPs was further enhanced under laser irradiation (Figure 6C–E). As shown in Figure 6E, the occlusive vessel was almost completely relieved by FT‐DT NPs under laser irradiation. The potent antithrombotic efficacy of FT‐DT NPs should be attributed to the photothermal‐amplified deep thrombus penetration and synergistic thrombolysis of TGL and DiR. Notably, there was no significant damage to the nearby blood vessel after treatment with FT‐DT NPs under laser irradiation (Figure S13, Supporting Information), suggesting the favorable therapeutic safety in vivo. Moreover, FT‐DT NPs did not induce erythrocyte hemolysis (Figure S14, Supporting Information), and there is no noticeable change observed in hepatorenal indicators and histological tissue sections (Figures S15,S16, Supporting Information). These results indicated that FT‐DT NPs had potent antithrombotic efficacy without significant off‐target toxicity to blood cells and major organs.

## 3 Conclusions

In the present study, a facile and practical co‐delivery strategy was elaborately developed to tackle several major challenges in antithrombotic therapy, including i) rapid clearance of therapeutic agents from the blood; ii) inadequate drug accumulation in the thrombi; iii) non‐intelligent drug release in systemic circulation; iv) insufficient deep permeability into the clots; and (v) unsatisfactory thrombolysis efficiency with high bleeding risks. Interestingly, we found that DiR and TGL readily co‐assemble into stable DT NPs driven by multiple intermolecular interactions and forces, including hydrophobic force, *π*–*π* stacking and *π*–cation interactions. Moreover, a PEGylated thrombus‐targeting nanoassembly (FT‐DT NPs) with the optimal DiR/TGL ratio (1:1, w/w) was fabricated by decorating the nanoassembly with DSPE‐PEG_2K_ and DSPE‐PEG_2K_‐CREKA. As expected, FT‐DT NPs demonstrated multiple advantages, including facile fabrication, high drug‐loading efficiency (37.5 wt% for both DiR and TGL), long circulation time in the blood, favorable thrombus‐targeted biodistribution, photothermal‐promoted thrombus deep penetration, as well as potent photothermal thrombolysis and antiplatelet efficiency. These advantages result in significantly photothermal‐amplified antithrombotic efficacy in vivo, with the thrombus blots almost completely disappearing in an FeCl_3_‐induced rat carotid arterial thrombosis model. Such a uniquely engineered dual‐drug nanoassembly has great potential to be used as potent multimodal thrombolysis nanomedicine in clinical thrombus therapy.

## Conflict of Interest

The authors declare no conflict of interest.

## Supporting information

Supporting Information

## Data Availability

Research data are not shared.
