# Supplementary material for: Elaborately Engineering a Self‐Indicating Dual‐Drug Nanoassembly for Site‐Specific Photothermal‐Potentiated Thrombus Penetration and Thrombolysis
Source: Adv Sci (Weinh). 2021 Nov 21;9(4):2104264. doi: 10.1002/advs.202104264 (PMC8811805; doi:10.1002/advs.202104264)
Supplement: Supplementary file 1 — Supporting Information [file ADVS-9-2104264-s001.pdf]

ADVANCED SCIENCE
Open Access

Supporting Information

for *Adv. Sci.*, DOI: 10.1002/advs.202102460

Elaborately Engineering a Self-indicating Dual-drug Nanoassembly for Site-specific Photothermal-potentiated Thrombus Penetration and Thrombolysis

Zhiqiang Zhao[a], Xuanbo Zhang[a], Hongyuan Zhang, Xinzhu Shan, Meiyu Bai, Zhe Wang, Fujun Yang, Haotian Zhang, Qiming Kan, Bingjun Sun, Jin Sun, Zhonggui He, Cong Luo[*]

Supporting Information

Elaborately Engineering a Self-indicating Dual-drug Nanoassembly for Site-specific Photothermal-potentiated Thrombus Penetration and Thrombolysis

Zhiqiang Zhao[a], *Xuanbo Zhang*[a], *Hongyuan Zhang, Xinzhu Shan, Meiyu Bai, Zhe Wang, Fujun Yang, Haotian Zhang, Qiming Kan, Bingjun Sun, Jin Sun, Zhonggui He, Cong Luo*[*]

Z. Zhao, Dr. X. Zhang, H. Zhang, X. Shan, M. Bai, Z. Wang, F. Yang, Dr. B. Sun, Prof. J. Sun, Prof. Z. He, Prof. C. Luo

Department of Pharmaceutics, Wuya College of Innovation, Shenyang Pharmaceutical University, Shenyang, Liaoning, 110016, P. R. China

Dr. H. Zhang, Q. Kan

School of Life Science and Biopharmaceutics, Shenyang Pharmaceutical University, Shenyang, 110016, P.R. China

Corresponding author: Prof. C. Luo; E-mail: luocong@syphu.edu.cn

[a]These authors contributed equally to this work.

Experimental Section

1. Materials

DiR, TGL, coumarin-6 and RBC Lysis buffer (10×) were obtained from Meilun Biotech Co. Ltd. (Dalian, China). 1,2-distearoyl-sn-glycero-3-phosphoethanolamine-N-[methoxy(polyethyleneglycol)-2000] (DSPE-PEG_{2K}) was obtained from AVT (Shanghai) Pharmaceutical Tech Co., Ltd. DSPE-PEG_{2K}-CREKA was synthesized by Suzhou Premierbiochem Co. Ltd. Other chemical reagents were analytical grade.

2. Preparation and characterization of nanoassemblies

The naked nanoassembly (DT NPs), PEGylated nanoassembly (PEG-DT NPs) and PEGylated fibrin-homing nanoassembly (FT-DT NPs) were prepared using a one-step nano-precipitation approach [1]. Briefly, 10 mg of DiR and 10 mg of TGL were dissolved in absolute ethyl alcohol (1 mL), respectively. Then, series mixtures of the above DiR Sol and TGL Sol with different mass ratios (3:1, 2:1, 1:1, 1:2, 1:3) of DiR and TGL were dripped respectively into purified water (2 mL) under intense stirring (1500 rpm, 3min) to obtain DT NPs. The optimal formulation of DT NPs was screened out by evaluating the particle size and colloidal stability of the nanoassembly. Moreover, PEG-DT NPs was prepared by adding a methanol solution of DSPE-PEG_{2k} (15%, w/w) into optimal DT NPs colloidal solution with vigorous stirring (1500 rpm, 2 min). Similarly, a mixed methanol solution of DSPE-PEG_{2k} (15 wt%) and DSPE-PEG_{2k}-CREKA (10 wt%) was dripped into optimal DT NPs solution to prepare

the PEGylated fibrin-targeting FT-DT NPs with vigorous stirring (1500 rpm, 2 min). Finally, the organic solvent in these nanoassembly systems was removed in vacuum at 33 °C.

The hydrodynamic diameters and zeta potentials of DT NPs and FT-DT NPs were measured by a Zetasizer (Nano ZS, Malvern Co., UK). The morphology of DT NPs and FT-DT NPs was observed by Transmission electron microscopy (TEM) (HITACHI, HT7700, Japan) after staining with phosphotungstic acid (2%, w/v).

3. Ultraviolet and Fluorescence Spectra

The ultraviolet (UV) spectra of DiR Sol, TGL Sol, DT NPs and FT-DT NPs and the fluorescence spectra of DiR Sol, DT NPs and FT-DT NPs (DiR and TGL 25 μg/mL) were acquired at an equivalent DiR or TGL concentration of 25 μg/mL using Varioskan Flash multimode microreader (Thermo Scientific, USA).

4. Co-Assembly Simulation

Molecular docking simulation method was used to investigate the intermolecular interactions of DiR and TGL. Moreover, the Autodock Vina software was utilized to construct the 3-dimentional structures of DiR and TGL. The runtime environment and optimized parameters have corresponded with our previous study [2].

5. Stability

To evaluate the stability of nanoassemblies, DT NPs, PEG-DT NPs and FT-DT NPs (50 μg/mL of DiR and TGL) were incubated in pure water or PBS (pH 7.4) for 30 min. Furthermore, to further explore the stability of nanoassemblies in a simulated

physiological environment, PEG-DT NPs and FT-DT NPs were incubated in PBS (pH 7.4) containing 10% FBS for 12 h at 37 ℃. The particle sizes of PEG-DT NPs and FT-DT NPs were measured by a Zetasizer. Moreover, the drugs leakage from nanoassemblies during the incubation process was explored by an ultrafiltration method. Briefly, 1 mL of PEG-DT NPs or FT-DT NPs of (TGL and DiR of 1 mg/mL) were incubated in PBS (pH 7.4) containing 10% FBS (10 mL) at 37 ℃. At pre-set time intervals, the samples (1 mL) were taken out, and the unencapsulated drugs were removed from the nanoassemblies by passing through an ultrafiltration membrane with a retention molecular weight of 30 kDa after centrifugation (4000 r/min, 10 min). Then, the concentrations of TGL in the filtrates were determined by HPLC. The chromatographic condition was as follows: C18 chromatographic column (4.6 × 150 mm, 5 μm); mobile phase: acetonitrile: water = 50: 50. The flow rate was set to 1.0 mL/min; the wavelength was set to 254 nm for TGL detection (n=3). And the concentrations of DiR in the filtrates were determined by Varioskan Flash multimode microreader (Thermo Scientific, USA). Additionally, the long-term stability of DT NPs, PEG-DT NPs and FT-DT NPs (0.5 mg/mL) were explored by determining the particle sizes and zeta potentials of nanoassemblies stored at 4 °C for one week.

6. *In vitro* thrombus-targeting capacity

Platelet-rich plasma was acquired from Sprague-Dawley rats. Clot formation was induced by incubating the platelet-rich plasma (150 μL) with thrombin (0.1 U/μL) and $CaCl_2$ (0.3 M) in a 96-well assay plate for 90 min [3]. After thrombosis, PBS, DiR Sol,

DT NPs, PEG-DT NPs and FT-DT NPs with an equivalent concentration of DiR (0.5 mg/mL) and TGL (0.5 mg/mL) were added on the top of clots and incubated for 10 min, respectively. Finally, the clots were washed three times with fresh PBS (pH 7.4), and the fluorescent signals were measured using an *in vivo* imaging system (IVIS Lumina Series III) (n=3).

7. *In vitro* light-triggered TGL release

The release behaviors of TGL from FT-DT NPs were determined using the dialysis method, and PBS (pH=7.4) containing 20% ethanol was utilized as the release medium. The nanoassemblies (TGL 0.5 mg/mL) were placed into dialysis bags and were immersed into the release medium at 37 ℃. The laser-treated group was exposed to 808 nm laser irradiation (2.0 W/cm^2, 15 min) beforehand. At pre-set time points, 150 μL of the samples were taken out to determine the concentrations of TGL using HPLC, and the equivalent volume of fresh media was replenished.

8. *In vitro* shear-responsive TGL release

The release behaviors of TGL from FT-DT NPs were determined using the dialysis method, and PBS (pH=7.4) containing 20% ethanol was selected as the release medium. To simulate the high hemodynamic shear stress at the thrombus site, FT-DT NPs (TGL 0.5 mg/mL) and a small rotor were put into dialysis bags to exert a slight stirring effect (300 rpm) on the nanoassemblies. The dialysis bag without stirring was set as the control group. At pre-set time points, 150 μL of the samples were taken out

to determine the concentrations of TGL using HPLC, and equivalent volume of fresh media was replenished.

9. *In vitro* photothermal conversion efficiency

To investigate the *in vitro* photothermal conversion efficiency, PBS, DiR Sol, DT NPs and FT-DT NPs with an equivalent DiR concentration of 0.5 mg/mL were exposed to laser irradiation (808 nm, 2.0 W/cm^2) for 15 min. An infrared thermal imaging camera (Fotric 226) was used to measure the temperature variations (n=3).

10. *In vitro* photothermal thrombolysis

An artificial blood clot was prepared to evaluate the photothermal thrombolysis effect. In brief, platelet-poor plasma (100 μL), $CaCl_2$ (2.5 mM), and thrombin (1 U/mL) were mixed and incubated in EP tubes (1.5 mL) for 1 h at 37 °C [4]. Then, PBS, DiR Sol, DT NPs and FT-DT NPs with an equivalent DiR concentration of 0.5 mg/mL was added into the artificial blood clot under laser irradiation (808 nm, 2.0 W/cm^2), respectively. After irradiation for 15 min, the EP tubes were put upside down several times for preliminary evaluation of the thrombolysis effect. Moreover, the supernatants in tubes were collected, and the absorbance of fibrin in the supernatants was measured at 450 nm using a multimode microreader (Thermo Scientific, USA).

11. Photothermal-promoted thrombus deep penetration

To track the FT-NPs in thrombus, Coumarin 6 (C-6), a fluorescence dye, was applied to label FT-DT NPs and obtain C-6-FT-DT NPs. To prepare the C-6-FT-DT NPs, mixtures ethanol Sol of DiR (100 μL,10 mg/mL), TGL (100 μL,10 mg/mL) and C-6

(50 μL, 1mg/mL) were dripped into purified water (2 mL) under intense stirring (1500 rpm, 3min) and subsequent experiment processes were same as the preparation method of FT-DT NPs.

A small red thrombus model was prepared to evaluate the thrombus penetration ability of FT-DT. Fresh blood was collected from Sprague-Dawley rats without adding anticoagulants. Then 5 μL blood were added to the bottom of 200 μL EP tube and incubated at 37 ℃ for 3 h to induce thrombosis. The formed blood clot was incubated with free C-6 and C-6-FT-DT NPs (C-6, 25 μg/mL) of 5 μL at 37℃ for 30 min and C-6-FT-DT NPs were further exposed to 808 nm irradiation for 10 min at a power density of 2 W/cm^2. Finally, the fluorescence signal in the thrombus interior was observed by a confocal laser scanning microscope (CLSM, C2, Nikon, Japan).

12. *In vitro* antiplatelet activity

Blood samples acquired from Sprague-Dawley rats were centrifuged (4×10^3 rpm, 5 min) to obtain platelet-rich plasma. Then, the 50 μL of fresh plasma samples containing 10 μL of thrombin (0.1 U/μL) in antibody-coated microplates were incubated with TGL Sol, DT NPs and FT-DT NPs at various TGL concentrations (200, 50 and 20 μg/mL) at room temperature for 2 h, respectively. The expression levels of sCD40L were measured with an ELISA kit (Mlbio, Shanghai, China) by following the manufacturer’s instructions (n=3) [5].

13. Animal studies

All the animal protocols were conformed to the Animal Laboratory Ethics Committee of Shenyang Pharmaceutical University (accreditation number: No. 19169).

14. Tail bleeding assay

After anesthetization by intraperitoneal injection of a chloral hydrate (3.5%, 10 μL/g), C57 mice intravenously received TGL Sol, DT-NPs and FT-DT NPs with different dosage (TGL 0.60 mg/kg, 0.75 mg/kg and 0.90 mg/kg). A distal segment (1cm) of the tail was cut off using a surgical scissors at 2 h post-administration, and the wounded tail was immersed in saline at 37 °C. To evaluate the antiplatelet effect, the bleeding time and blood volume were recorded. Bleeding time refer to the time needed for a wound to stop bleeding for at least 10 s. To determine the bleeding volume, the blood samples were centrifuged at 4000g for 5 min, and the supernatants were discarded to obtain the blood cells. Then, the blood cells were resuspended in 2 mL of lysis buffer. The absorbance of hemoglobin in the lysates at 550 nm was analyzed using a multimode microreader (Thermo Scientific, USA). The bleeding volume was evaluated by comparing the measured ultraviolet absorption to that of the standard blood samples (n=5) [6].

Moreover, to further verify the targeting ability of FT-DT NPs, the hemostatic amputated tails were obtained and soaked into DT NPs and FT-DT NPs for 3 min. Then, the amputated tails were taken out and washed by PBS three times, and their fluorescent signal was detected using an *in vivo* imaging system (IVIS Lumina Series III).

15. Pharmacokinetics

To investigate the pharmacokinetics behaviors of co-delivery nanoassemblies, DiR Sol, TGL Sol, DT NPs, and FT-DT NPs were intravenously injected into Sprague-Dawley rats (180-220 g) at an equivalent dose with DiR (5 mg/kg) and TGL (5 mg/kg), respectively (n=5). At predesigned 0.05, 0.167, 0.5, 1, 2, 4, 6, 8, 12 and 24 h, about 500 µL of blood were harvested from each rat and centrifuged (6000 rpm, 2 min) to obtain the plasma. The protein precipitation method was employed to extract DiR and TGL from the plasma. Then, the plasma concentrations of DiR and TGL were detected by using multimode microreader (Thermo Scientific, USA) and UPLC-MS-MS (AB SCIEX Corp., USA), respectively.

16. Thrombus-targeting fluorescence imaging

Sprague-Dawley rats (180-220 g) were anaesthetized by intraperitoneal injection of chloral hydrate (3.5%, 10 µL/g). After scraping the fur on the neck, the rats were placed and fixed on a surgical table using strings. To expose the carotid arteries, a midline incision was made between the chin and sternum, and the peripheral muscles were separated using fine forceps. Then, a filter paper (10 × 10 mm) saturated with 10% $FeCl_3$ was wrapped on the right carotid artery for 10 min to thrombosis. Finally, PBS was used to wash the residual $FeCl_3$ for three times [7]. After thrombus formation, DiR Sol, DT NPs and FT-DT NPs were intravenously injected into rats at a DiR equivalent dose of 5 mg/kg, respectively. The embolic vessel fluorescence images were observed

using a noninvasive optical imaging system (IVIS Lumina Series III) at 5, 15, 30, 60 and 90 min post-administration, respectively (n=5).

17. *In vivo* photothermal efficacy

A $FeCl_3$-induced arterial thrombosis rat model was established by using the same method described previously. To investigated the *in vivo* photothermal efficiency, PBS, DiR Sol, DT NPs, and FT-DT NPs were intravenously injected into rats at a DiR equivalent dose of 5 mg/kg, respectively. The rat necks were exposed to laser (808 nm, 2 W/cm^2) for 15 min at 1 h post-administration (n=5). The infrared thermographic images and local temperature variations were recorded by infrared thermal imaging camera (Fotric 226).

18. *In vivo* photothermal-facilitated antithrombic effect

The Sprague-Dawley rats (180-220 g) were randomly divided into ten groups: 1) untreated group; 2) embolized group; 3) laser group; 4) DiR Sol group; 5) TGL Sol group; 6) DiR Sol + laser group; 7) DT NPs group; 8) DT NPs + laser group; 9) FT-DT NPs group; 10) FT-DT NPs + laser group (n=6). The drug-treated rats received an equivalent dosage of DiR and/or TGL (5mg/kg). As previously described, a $FeCl_3$-induced arterial thrombosis rat model was established by using the same method. The neck of rats was exposed to laser (808 nm, 2.0 W/cm^2) for 15 min at 1 h post-administration. One hour later, the rats were sacrificed, and the carotid vessels were harvested and dried for measurement of the dry weight of the thrombus. (Thrombus therapy rate = (thrombus weight before therapy-thrombus weight after therapy)/

thrombus weight before therapy) The photographs of carotid artery embolization in rats with or without FT-DT NPs treatment under laser irradiation were recorded. In addition, the embolismic vessels were washed by PBS and fixed in the 4% paraformaldehyde, and stained with hematoxylin and eosin (H&E) to evaluate the thrombus treatment effect.

19. Preliminary evaluation of therapeutic safety

For hemolysis evaluation, the collected erythrocytes (50 μL) were diluted into PBS (1 mL). Then the TGL Sol, DiR Sol, DT NPs and FT-DT NPs (50 μL) with the same concentrations (DiR and TGL 0.1 mg/mL) were added into erythrocytes PBS Sol and then kept at 37 °C for 3 h, followed by centrifugation at 10000 rpm for 3 min (n=3). Finally, the supernatant was collected and its absorbance was measured at 540 nm with PBS (pH 7.4) as the negative control and pure water as the positive control.

Healthy Sprague-Dawley rats were intravenously administrated with DT NPs and FT-DT NPs (5 mg/kg for both DiR and TGL), and the neck of rats was exposed to laser (808 nm, 2.0 W/cm^2) for 15 min at 1 h post-administration. One day later, the plasma was collected for hepatic and renal function measurements (n=5). Moreover, the main organs (heart, liver, spleen, lung and kidney) were washed by PBS, fixed in the 4% paraformaldehyde, and stained with H&E to evaluate the pathological changes.

20. Statistical analysis

The data were calculated and expressed as mean value ± standard deviation. Student's T-test and one-way analysis of variance (ANOVA) were employed to analyze the differences. The difference was considered as statistically significant when the p values were less than 0.05.

Supporting Figures

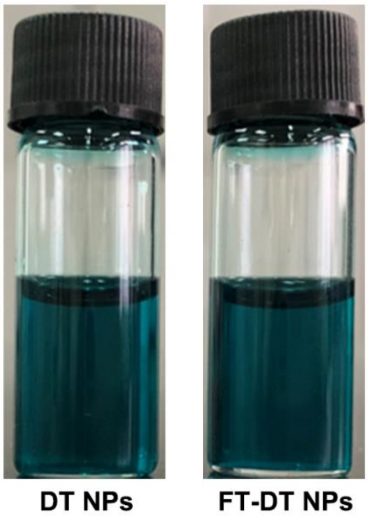

Figure S1. Photographs of DT NPs and FT-DT NPs.

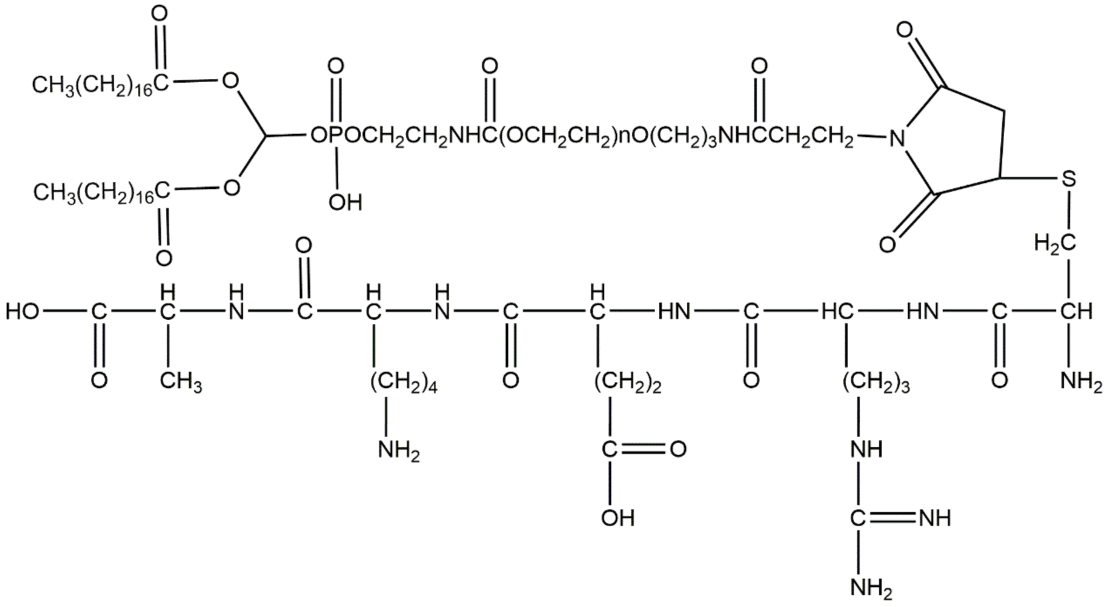

Figure S2. Chemical structure of DSPE-PEG-CREKA.

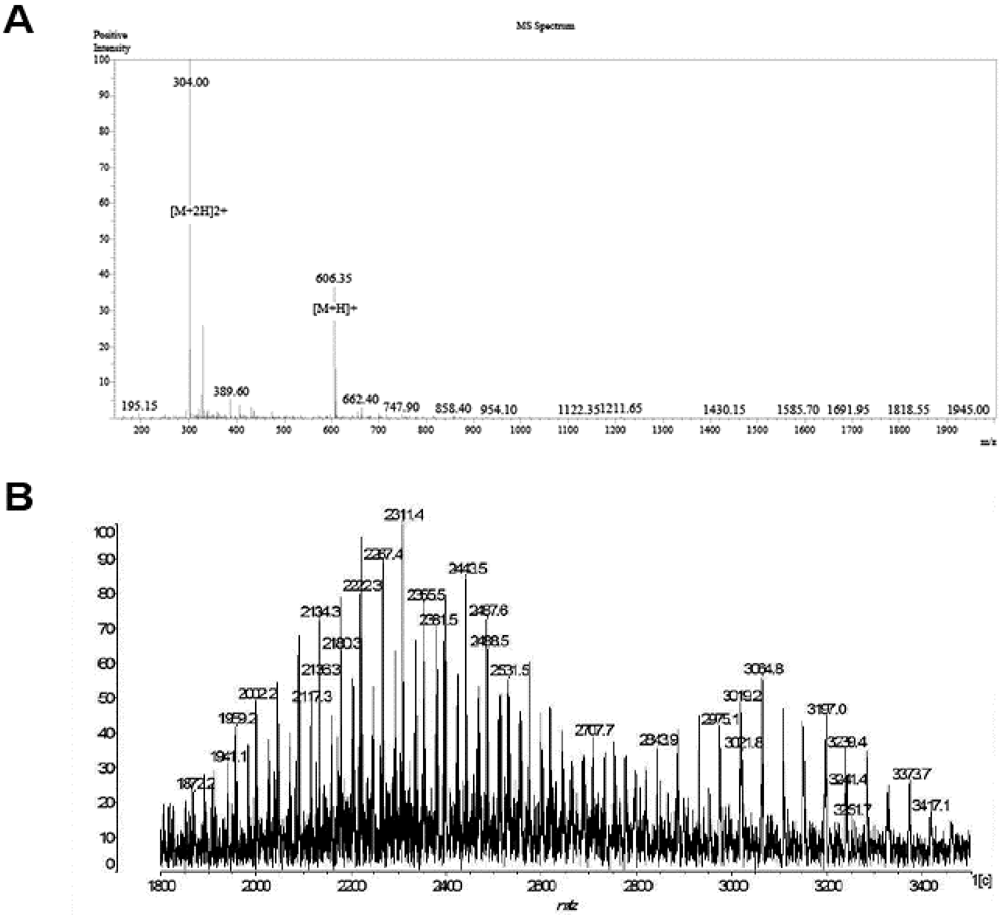

Figure S3. MS of CREKA (Cys-Arg-Glu-Lys-Ala) and DSPE-PEG-CREKA. (A) CREKA; (B) DSPE-PEG-CREKA.

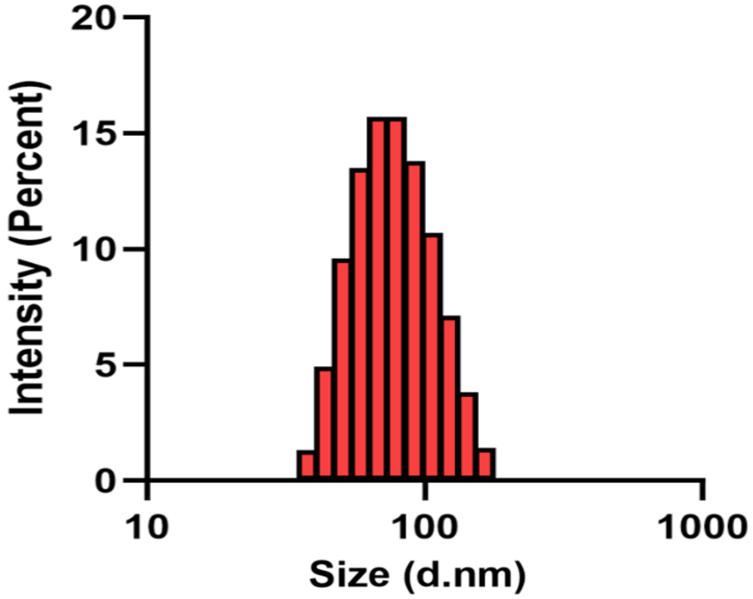

Figure S4. Particle size of PEG-DT NPs.

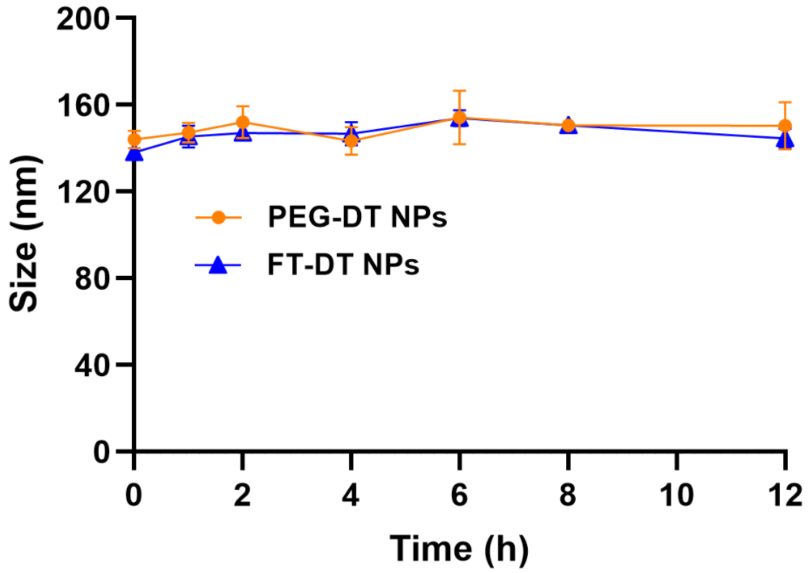

Figure S5. Colloidal stability of PEG-DT NPs and FT-DT NPs incubated in PBS (pH 7.4) containing 10% FBS for 12 h at 37 ℃.

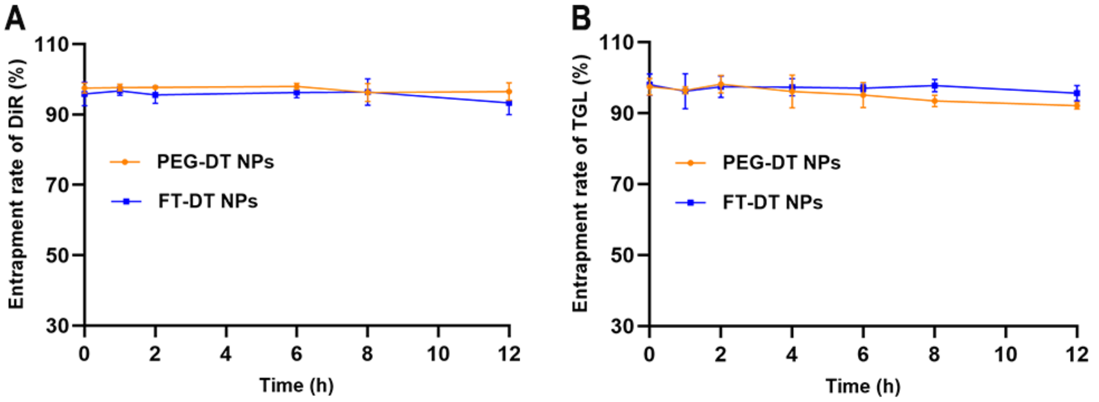

Figure S6. The remaining of DiR (A) and TGL (B) in PEG-DT NPs and FT-DT NPs incubated in PBS (pH 7.4) containing 10% FBS for 12 h at 37 ℃.

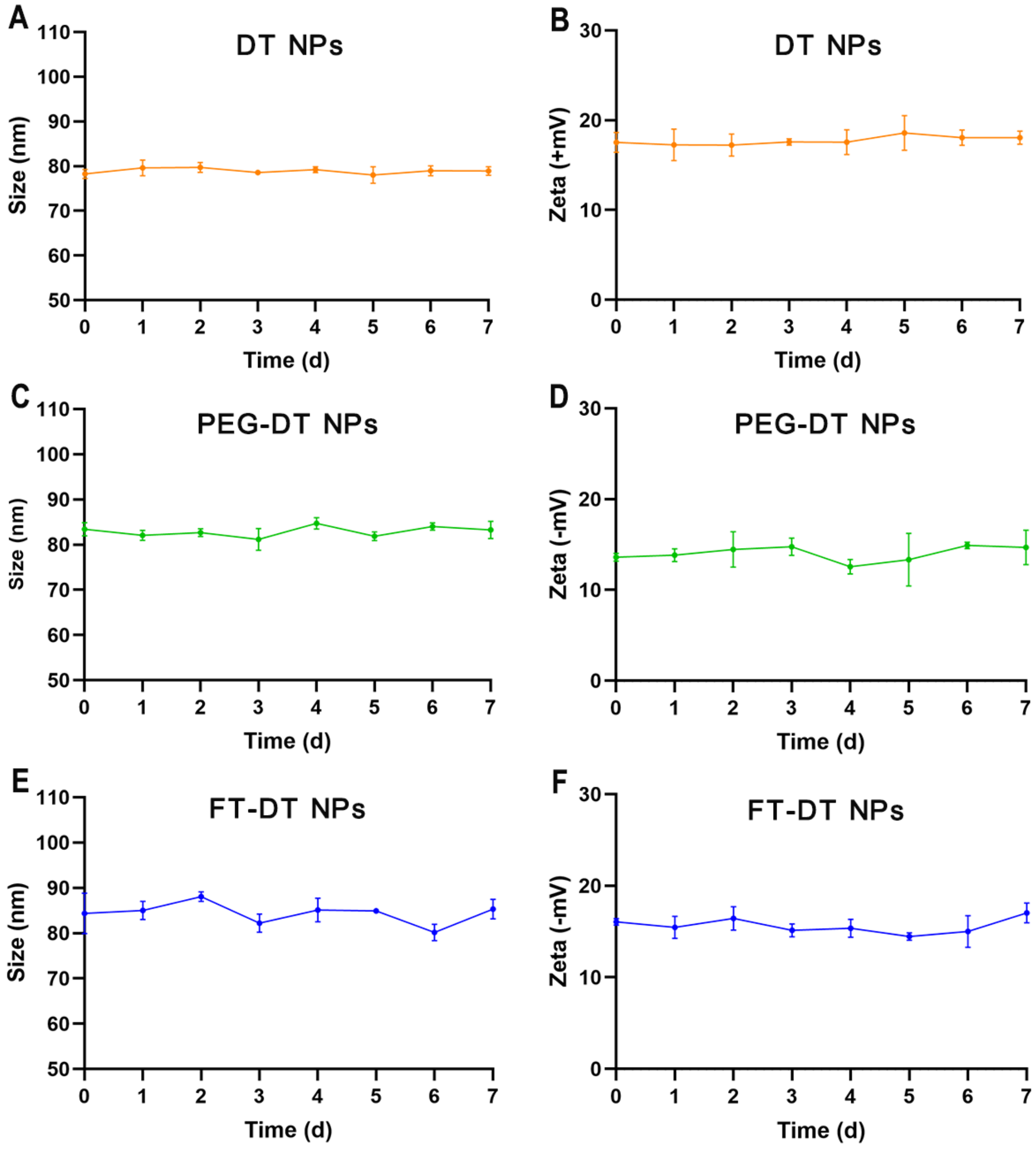

Figure S7. Long-term storage stability of DT NPs, PEG-DT NPs and FT-DT NPs after storage at 4 °C for one week. (A) Particle sizes of DT NPs. (B) Zeta potentials of DT NPs. (C) Particle sizes of PEG-DT NPs. (D) Zeta potentials of PEG-DT NPs. (E) Particle sizes of FT-DT NPs. (F) Zeta potentials of FT-DT NPs.

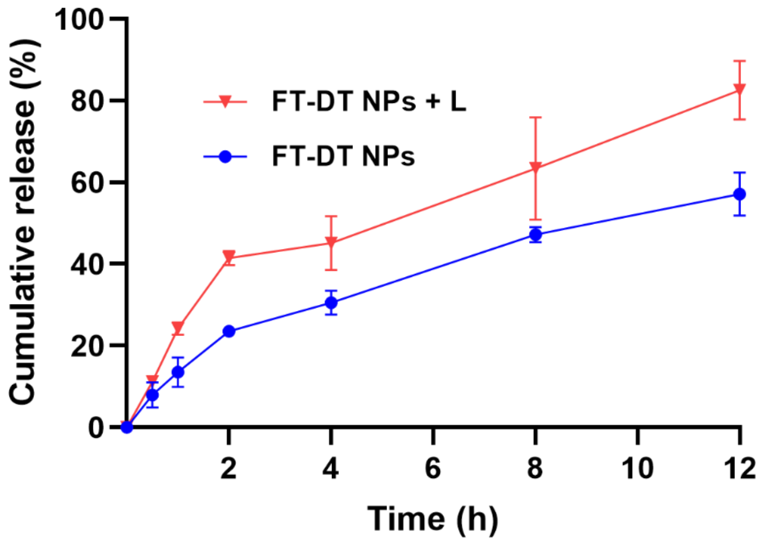

Figure S8. Cumulative release of TGL from FT-DT NPs with or without laser irradiation (808 nm, 2.0 W/cm^2, 15 min) for 12 h.

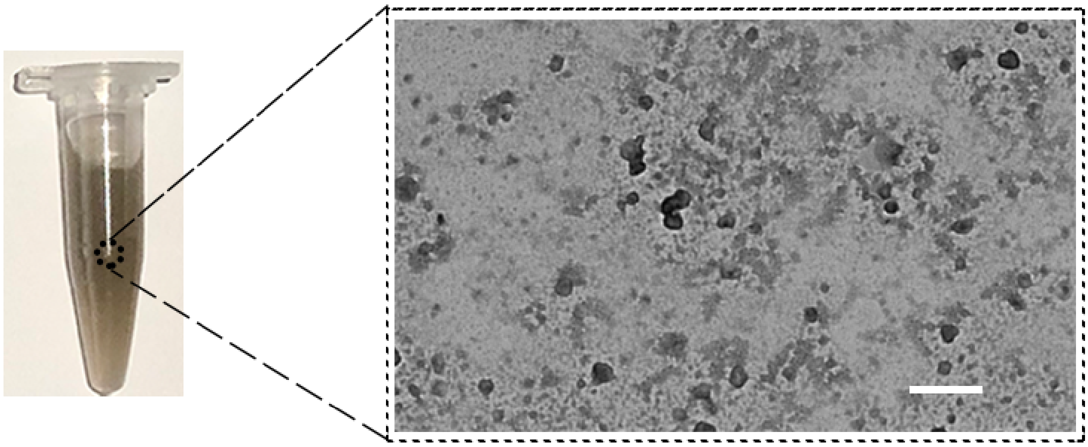

Figure S9. TEM image of FT-DT NPs post irradiation (808 nm, 2.0 W/cm^2, 15 min). Scale bar represents 500 nm.

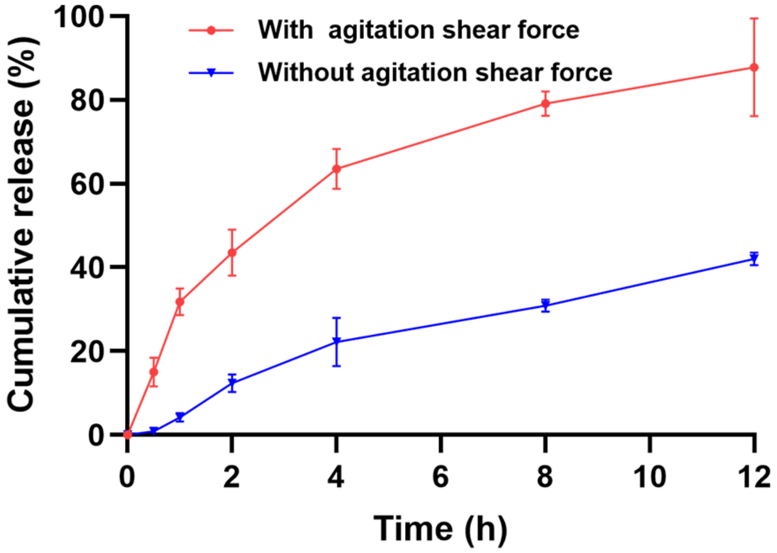

Figure S10. The release behaviors of TGL from FT-DT NPs *in vitro* with or without agitation shear force (300 rpm) for 12 h.

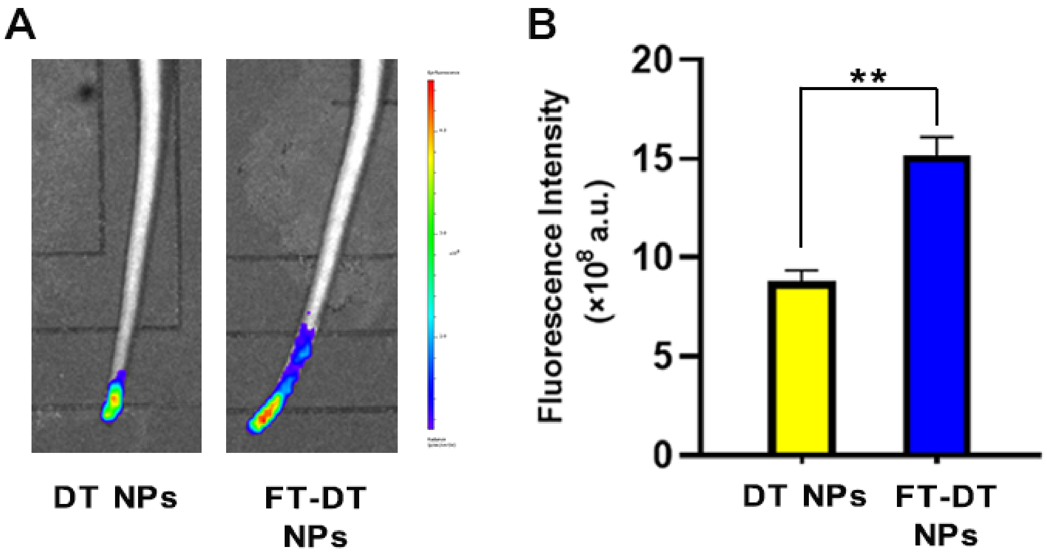

Figure S11. (A) Fluorescence images of transected tails of mice soaked with DT NPs or FT-DT NPs. (B) Quantitative analysis of fluorescence intensity of transected tails. (n=5) **p<0.01.

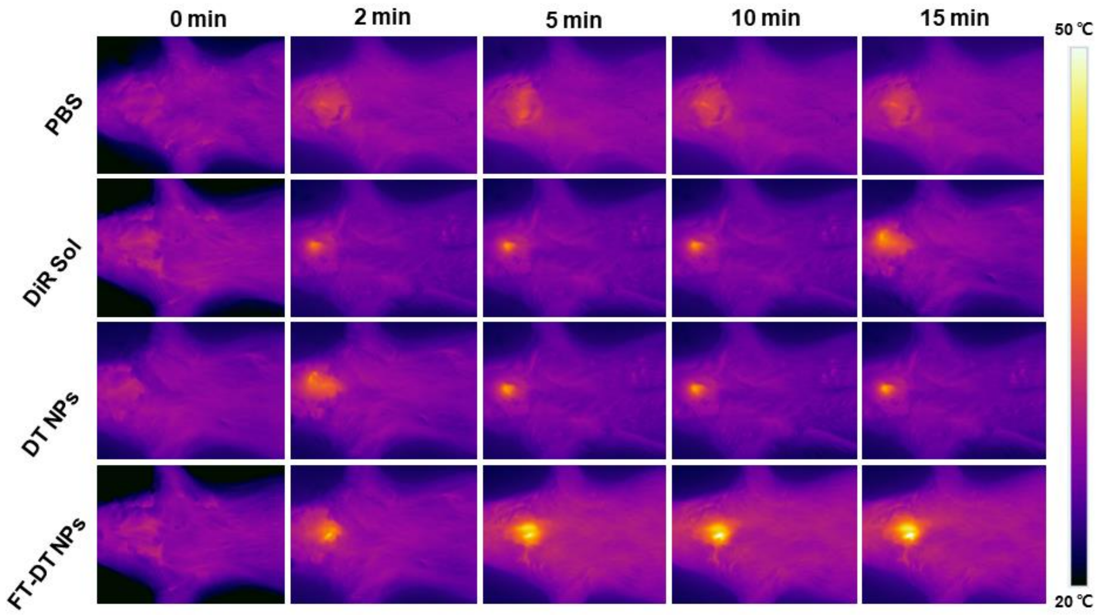

Figure S12. *In vivo* photothermal imaging of thrombus location under 808 nm laser treatment (2.0 W/cm ^{2}, 0-15min).

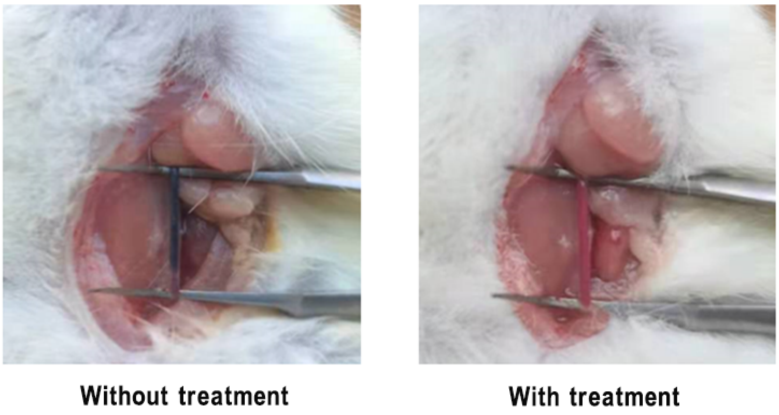

Figure S13. Photographs of the carotid artery embolization in rats with or without FT-DT NPs treatment under laser irradiation (808 nm, 2.0 W/cm^2, 15 min).

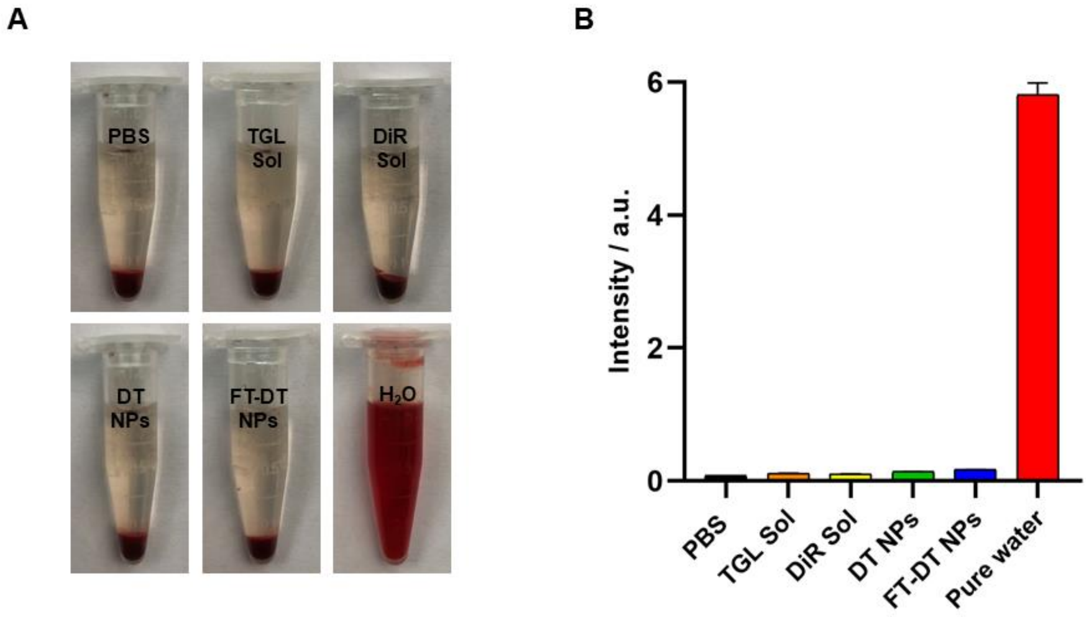

Figure S14. Hemolysis evaluation of TGL Sol, DiR Sol, DT NPs and FT-DT NPs with an equivalent DiR and TGL concentration. (0.1 mg/mL) (A) Hemolysis photograph of above groups. (B) Quantitative analysis of hemoglobin content of supernatant (n=3).

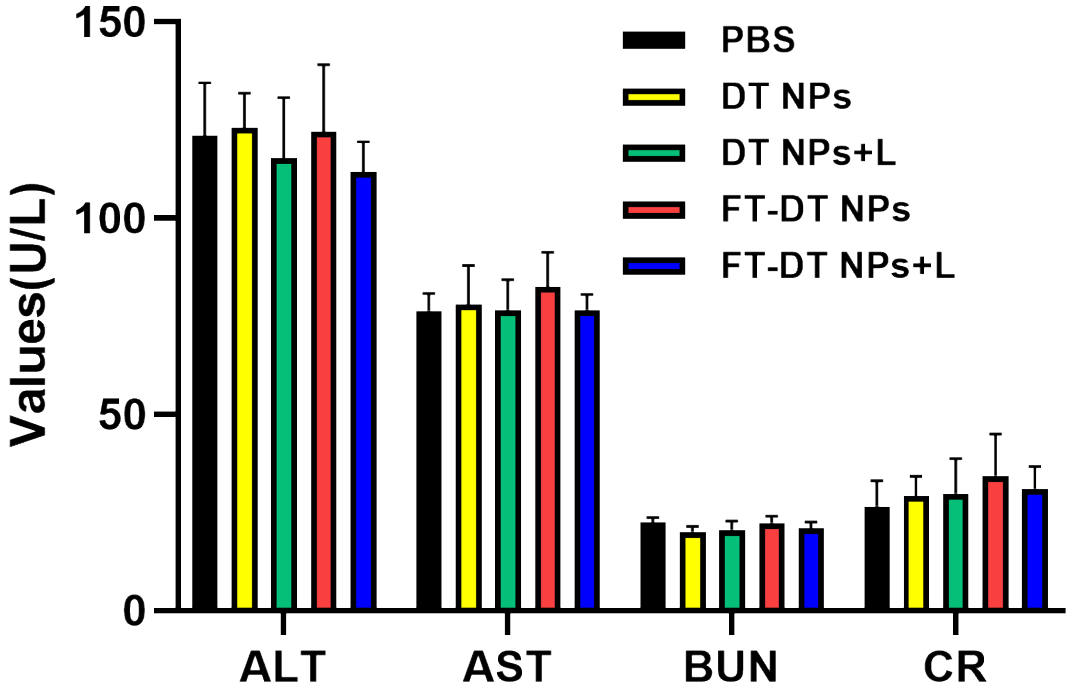

Figure S15. Hepatorenal function parameters of rats (n=5). (ALT: alanine aminotransferase, AST: aspartate aminotransferase, BUN: blood urea nitrogen, CR: creatinine).

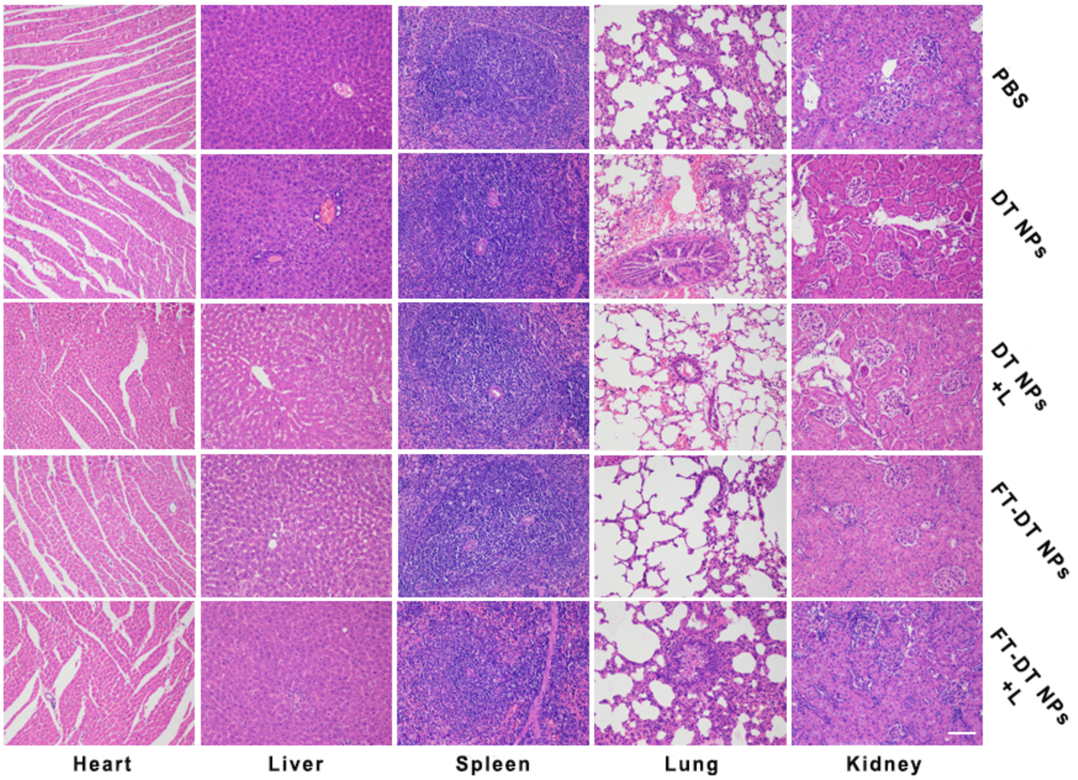

Figure S16. H&E staining of heart, liver, spleen, lung and kidney of rats. Scale bar represents 100 μm.

Supporting Tables

Table S1. Characteristics of DT NPs with different prescriptions.

Formulations	Size[a)] (nm)	PDI[b)]
3:1	112.70 ± 1.92	0.38 ± 0.04
2:1	90.06 ± 3.50	0.15 ± 0.03
1:1	74.96 ± 0.42	0.14 ± 0.05
1:2	81.52 ± 3.31	0.23 ± 0.01
1:3	84.89 ± 10.33	0.19 ± 0.02

a) Mean diameters of DT NPs determined by DLS. b) Polydispersity index of DT NPs.

Table S2. Characteristics of DT NPs and FT-DT NPs.

Nanoassemblies	Size[a)] (nm)	PDI[b)]	Zeta[a)] (mV)	Drug loading rate[c)]
DT-NPs	74.96 ± 0.42	0.14 ± 0.05	+17.50 ± 0.09	DiR or TGL=50 %
PEG-DT NPs	78.39±1.62	0.19±0.03	-16.20±1.36	DiR or TGL=42.5 %
FT-DT NPs	80.26 ± 1.25	0.19 ± 0.02	-16.10 ± 0.35	DiR or TGL=37.5 %

a) Mean diameters and Zeta potential of nanoassemblies determined by DLS. b) Polydispersity index of nanoassemblies. c) Drug loading rate of nanoassemblies.

Table S3. Pharmacokinetic parameters of DiR in DiR Sol, DT NPs and FT NPs. (n=5)

Formulations	$C_{0.05}$ [a)]	$AUC_{0\text{-}24h}$ [b)]
DiR Sol	1.42 ± 0.61	13.36 ± 1.35
DT NPs	4.51 ± 4.93	19.73 ± 1.30
FT-DT NPs	21.25 ± 2.55	102.6 ± 4.96

a) The plasma concentrations at 0.05 h (μg/mL). b) Area under the plasma concentration time curve (μg/mL*h).

Table S4. Pharmacokinetic parameters of TGL in TGL Sol, DT NPs and FT NPs. (n=5)

Formulations	$C_{0.05}$ a)	$AUC_{0\text{-}24h}$ b)
TGL Sol	1.70 ± 0.68	2.53 ± 0.20
DT NPs	1.77 ± 0.39	3.14 ±1.32
FT-DT NPs	14.50 ± 1.34	18.66 ± 1.03

The plasma concentrations at 0.05 h (μg/mL). b) Area under the plasma concentration time curve (μg/mL*h).

Supporting References

[1] X. Zhang, B. Sun, S. Zuo, Q. Chen, Y. Gao, H. Zhao, M. Sun, P. Chen, H. Yu, W. Zhang, K. Wang, R. Zhang, Q. Kan, H. Zhang, Z. He, C. Luo, J. Sun, *ACS Appl. Mater. Interfaces*. **2018**, 10, 30155.

[2] a) Q. Wang, M. C. Sun, D. Li, C. Li, C. Luo, Z. M. Wang, W. J. Zhang, Z. M. Yang, Y. Feng, S. Wang, Z. G. He, H. T. Zhang, Q. M. Kan, W. Sun, J. Sun, *Theranostics*. **2020**, *10*, 5550; b) S Zhang, Z Wang, Z Kong, Y Wang, X Zhang, B Sun, H Zhang, Q Kan, Z He, C Luo, J Sun, *Theranostics* **2021**, *11*, 6019.

[3] J. Lee, L. Jeong, E. Jung, C. Ko, S. Seon, J. Noh, D. Lee, *J. Control. Release* **2019**, *304*, 164.

[4] Nitesh Singh, Anand Varma, Ashish Verma, Babu N. Maurya, Debabrata Dash, *Nano Res*. **2016**, *9*, 2327.

[5] C. Kang, S. Gwon, C. Song, P. M. Kang, S. C. Park, J. Jeon, D. W. Hwang, D. Lee, *ACS Nano* **2017**, *11*, 6194.

[6] E. Jung, C. Kang, J. Lee, D. Yoo, D. W. Hwang, D. Kim, S. C. Park, S. K. Lim, C. Song, D. Lee, *ACS Nano* **2018**, *12*, 392.

[7] a) K. D. Kurz, B. W. Main, G. E. Sandusky, *Thromb. Res*. **1990**, *60*, 269; b) X. Wang, L. Xu, *Thromb. Res*. **2005**, *115*, 95.
